# (4,5-Diaza­fluoren-9-one-κ^2^ *N*,*N*′)bis­(thio­cyanato-κ*S*)mercury(II)

**DOI:** 10.1107/S1600536811008142

**Published:** 2011-03-09

**Authors:** Behrouz Notash, Nasser Safari, Vahid Amani

**Affiliations:** a Department of Chemistry, Shahid Beheshti University, G. C., Evin, Tehran 1983963113, Iran

## Abstract

In the title compound, [Hg(NCS)_2_(C_11_H_6_N_2_O)], the Hg^II^ atom, lying on a twofold rotation axis, is four-coordinated in a distorted tetra­hedral geometry by an *N*,*N*′-bidentate diaza­fluoren-9-one ligand and two thio­cyanate anions. In the crystal, inter­molecular C—H⋯N and C—H⋯O hydrogen bonds are effective in the stabilization of the structure.

## Related literature

For general background to metal complexes with diaza­fluoren-9-one ligands, see: Biju & Rajasekharan (2008 ▶); Kulkarni *et al.* (2002 ▶); Menon & Rajasekharan (1998 ▶); Shi *et al.* (1995 ▶); Wu & Xu (2004 ▶); Zhang *et al.* (2004 ▶). For related structures, see: Ravikumar & Lakshmi (1994 ▶); Safari *et al.* (2009 ▶). For the synthesis of the ligand, see: Henderson *et al.* (1984 ▶).
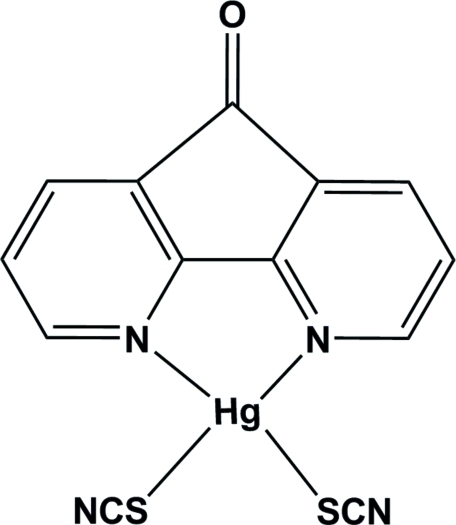


## Experimental

### 

#### Crystal data

[Hg(NCS)_2_(C_11_H_6_N_2_O)]
*M* *_r_* = 498.95
Monoclinic, 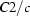

*a* = 10.570 (2) Å
*b* = 16.112 (3) Å
*c* = 8.3390 (17) Å
β = 94.35 (3)°
*V* = 1416.1 (5) Å^3^
*Z* = 4
Mo *K*α radiation
μ = 11.17 mm^−1^
*T* = 298 K
0.45 × 0.30 × 0.25 mm

#### Data collection

Stoe IPDS-2T diffractometer
Absorption correction: numerical (*X-SHAPE* and *X-RED32*; Stoe & Cie, 2005 ▶) *T*_min_ = 0.023, *T*_max_ = 0.059
4787 measured reflections
1903 independent reflections
1737 reflections with *I* > 2σ(*I*)
*R*_int_ = 0.098

#### Refinement

*R*[*F* ^2^ > 2σ(*F* ^2^)] = 0.048
*wR*(*F* ^2^) = 0.118
*S* = 1.09
1903 reflections
97 parameters
H-atom parameters constrained
Δρ_max_ = 4.61 e Å^−3^
Δρ_min_ = −1.82 e Å^−3^

### 

Data collection: *X-AREA* (Stoe & Cie, 2005 ▶); cell refinement: *X-AREA*; data reduction: *X-AREA*; program(s) used to solve structure: *SHELXS97* (Sheldrick, 2008 ▶); program(s) used to refine structure: *SHELXL97* (Sheldrick, 2008 ▶); molecular graphics: *ORTEP-3* (Farrugia, 1997 ▶); software used to prepare material for publication: *WinGX* (Farrugia, 1999 ▶).

## Supplementary Material

Crystal structure: contains datablocks I, global. DOI: 10.1107/S1600536811008142/hy2412sup1.cif

Structure factors: contains datablocks I. DOI: 10.1107/S1600536811008142/hy2412Isup2.hkl

Additional supplementary materials:  crystallographic information; 3D view; checkCIF report

## Figures and Tables

**Table 1:** Selected bond lengths (Å)

Hg1—S1	2.4098 (17)
Hg1—N1	2.483 (5)

**Table 2:** Hydrogen-bond geometry (Å, °)

*D*—H⋯*A*	*D*—H	H⋯*A*	*D*⋯*A*	*D*—H⋯*A*
C2—H2⋯N2^i^	0.93	2.56	3.276 (10)	134
C4—H4⋯O1^ii^	0.93	2.59	3.366 (8)	142

Symmetry codes: (i) 
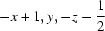
; (ii) 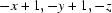.

**Fig. 1.**
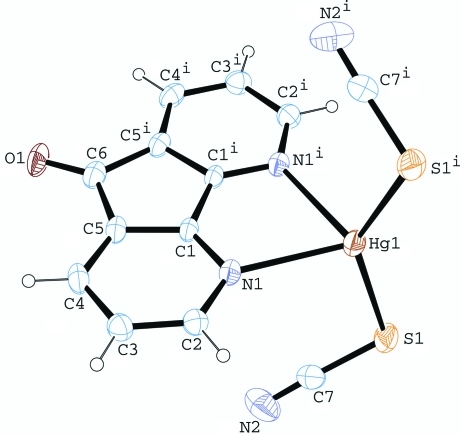
The molecular structure of the title compound, with displacement ellipsoids drawn at the 30% probability level. [Symmetry code: (i) -x+1, y, -z+1/2.]

**Fig. 2.**
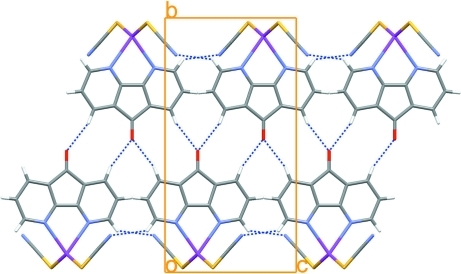
The packing diagram of the title compound. Intermolecular C—H···O and C—H···N hydrogen bonds are shown as blue dashed lines.

## supplementary crystallographic information

### Comment

Henderson *et al.* (1984) first reported the synthesis of diazafluoren-9-one (dafone) and Ravikumar & Lakshmi (1994) determined the structure of this compound. Dafone is a bidentate ligand and numerous complexes with dafone have been prepared, such as that of cobalt (Shi *et al.*, 1995), copper (Kulkarni *et al.*, 2002; Menon & Rajasekharan, 1998), zinc (Zhang *et al.*, 2004), manganese (Wu & Xu, 2004) and silver (Biju & Rajasekharan, 2008). For further investigation of the dafone complexes, we synthesized the title compound.

The asymmetric unit of the title compound (Fig. 1) contains a half molecule. The Hg^II^ atom, lying on a twofold rotation axis, is four-coordinated in a distorted tetrahedral geometry by an N,N'-bidentate dafone ligand and two thiocyanate anions. The Hg—N and Hg—S bond lengths (Table 1) and angles are within normal range as observed in [Hg(SCN)_2_(dm4bt)] (dm4bt = 2,2'-dimethyl-4,4'-bi-1,3-thiazole) (Safari *et al.*, 2009). In the crystal, intermolecular C—H···O and C—H···N hydrogen bonds stabilize the structure (Table 2, Fig. 2).

### Experimental

For the preparation of the title compound, a solution of dafone (0.13 g, 0.71 mmol) in methanol (20 ml) was added to a solution of Hg(SCN)_2_ (0.23 g, 0.71 mmol) in methanol (20 ml) at room temperature and the yellow powder was formed. Crystals suitable for X-ray diffraction were obtained by methanol vapor diffusion into a DMSO solution of the complex. The crystals were isolated after one week (yield: 0.28 g, 79%).

### Refinement

H atoms were positioned geometrically and refined as riding atoms, with C—H = 0.93 Å and *U*_iso_(H) = 1.2*U*_eq_(C). The highest residual electron density was found at 0.90 Å from Hg1 atom and the deepest hole at 0.84 Å from Hg1 atom.

### Crystal data

**Table:**

[Hg(NCS)_2_(C_11_H_6_N_2_O)]	*F*(000) = 928
*M**_r_* = 498.95	*D*_x_ = 2.340 Mg m^−^^3^
Monoclinic, *C*2/*c*	Mo *K*α radiation, λ = 0.71073 Å
Hall symbol: -C 2yc	Cell parameters from 1903 reflections
*a* = 10.570 (2) Å	θ = 2.3–29.1°
*b* = 16.112 (3) Å	µ = 11.17 mm^−^^1^
*c* = 8.3390 (17) Å	*T* = 298 K
β = 94.35 (3)°	Block, yellow
*V* = 1416.1 (5) Å^3^	0.45 × 0.30 × 0.25 mm
*Z* = 4	

### Data collection

**Table:**

Stoe IPDS-2T diffractometer	1903 independent reflections
Radiation source: fine-focus sealed tube	1737 reflections with *I* > 2σ(*I*)
graphite	*R*_int_ = 0.098
ω scans	θ_max_ = 29.1°, θ_min_ = 2.3°
Absorption correction: numerical (*X-SHAPE* and *X-RED32*; Stoe & Cie, 2005)	*h* = −14→14
*T*_min_ = 0.023, *T*_max_ = 0.059	*k* = −19→22
4787 measured reflections	*l* = −11→9

### Refinement

**Table:**

Refinement on *F*^2^	Primary atom site location: structure-invariant direct methods
Least-squares matrix: full	Secondary atom site location: difference Fourier map
*R*[*F*^2^ > 2σ(*F*^2^)] = 0.048	Hydrogen site location: inferred from neighbouring sites
*wR*(*F*^2^) = 0.118	H-atom parameters constrained
*S* = 1.09	*w* = 1/[σ^2^(*F*_o_^2^) + (0.0771*P*)^2^] where *P* = (*F*_o_^2^ + 2*F*_c_^2^)/3
1903 reflections	(Δ/σ)_max_ < 0.001
97 parameters	Δρ_max_ = 4.61 e Å^−^^3^
0 restraints	Δρ_min_ = −1.81 e Å^−^^3^

### Fractional atomic coordinates and isotropic or equivalent isotropic displacement parameters (Å^2^)

**Table:**

	*x*	*y*	*z*	*U*_iso_*/*U*_eq_	
C7	0.7118 (6)	0.0950 (5)	0.0048 (8)	0.0493 (13)	
Hg1	0.5000	0.072315 (18)	0.2500	0.04567 (15)	
N1	0.4449 (4)	0.1960 (3)	0.0815 (6)	0.0365 (9)	
C1	0.4721 (4)	0.2635 (3)	0.1663 (6)	0.0320 (9)	
C2	0.3956 (5)	0.2094 (4)	−0.0707 (7)	0.0422 (11)	
H2	0.3735	0.1637	−0.1350	0.051*	
C5	0.4558 (5)	0.3449 (3)	0.1142 (7)	0.0377 (10)	
C3	0.3766 (6)	0.2879 (4)	−0.1349 (7)	0.0453 (12)	
H3	0.3438	0.2937	−0.2409	0.054*	
C4	0.4063 (5)	0.3591 (4)	−0.0419 (8)	0.0456 (12)	
H4	0.3935	0.4124	−0.0827	0.055*	
S1	0.68555 (17)	0.01847 (11)	0.1348 (2)	0.0576 (4)	
O1	0.5000	0.4758 (4)	0.2500	0.0586 (17)	
C6	0.5000	0.4016 (6)	0.2500	0.0432 (17)	
N2	0.7356 (8)	0.1448 (6)	−0.0831 (10)	0.083 (2)	

### Atomic displacement parameters (Å^2^)

**Table:**

	*U*^11^	*U*^22^	*U*^33^	*U*^12^	*U*^13^	*U*^23^
C7	0.049 (3)	0.058 (4)	0.041 (3)	0.004 (3)	0.005 (2)	−0.002 (3)
Hg1	0.0541 (2)	0.03049 (19)	0.0543 (2)	0.000	0.01618 (14)	0.000
N1	0.0374 (19)	0.029 (2)	0.043 (2)	0.0004 (16)	0.0032 (16)	0.0006 (17)
C1	0.0334 (19)	0.022 (2)	0.040 (3)	0.0006 (16)	0.0044 (17)	0.0012 (16)
C2	0.046 (3)	0.040 (3)	0.040 (3)	0.004 (2)	0.002 (2)	0.001 (2)
C5	0.035 (2)	0.028 (2)	0.050 (3)	0.0014 (18)	0.0046 (19)	0.005 (2)
C3	0.046 (3)	0.047 (3)	0.043 (3)	0.007 (2)	0.005 (2)	0.007 (2)
C4	0.046 (3)	0.035 (3)	0.056 (3)	0.005 (2)	0.006 (2)	0.015 (2)
S1	0.0623 (9)	0.0412 (8)	0.0715 (11)	0.0171 (7)	0.0201 (8)	0.0072 (7)
O1	0.072 (4)	0.023 (3)	0.081 (5)	0.000	0.007 (4)	0.000
C6	0.038 (3)	0.033 (4)	0.060 (5)	0.000	0.011 (3)	0.000
N2	0.077 (4)	0.104 (7)	0.070 (5)	0.003 (4)	0.021 (3)	0.025 (4)

### Geometric parameters (Å, °)

**Table:**

C7—N2	1.128 (11)	C2—H2	0.9300
C7—S1	1.679 (8)	C5—C4	1.385 (8)
Hg1—S1	2.4098 (17)	C5—C6	1.502 (8)
Hg1—N1	2.483 (5)	C3—C4	1.407 (9)
N1—C1	1.316 (7)	C3—H3	0.9300
N1—C2	1.352 (7)	C4—H4	0.9300
C1—C5	1.388 (6)	O1—C6	1.195 (12)
C1—C1^i^	1.473 (10)	C6—C5^i^	1.502 (8)
C2—C3	1.382 (8)		
			
N2—C7—S1	176.4 (8)	C3—C2—H2	118.5
S1^i^—Hg1—S1	137.80 (9)	C4—C5—C1	118.7 (5)
S1^i^—Hg1—N1^i^	103.08 (11)	C4—C5—C6	132.9 (5)
S1—Hg1—N1^i^	110.60 (12)	C1—C5—C6	108.4 (5)
S1^i^—Hg1—N1	110.60 (12)	C2—C3—C4	120.9 (5)
S1—Hg1—N1	103.08 (11)	C2—C3—H3	119.6
N1^i^—Hg1—N1	73.2 (2)	C4—C3—H3	119.6
C1—N1—C2	115.2 (5)	C5—C4—C3	115.8 (5)
C1—N1—Hg1	109.0 (3)	C5—C4—H4	122.1
C2—N1—Hg1	135.7 (4)	C3—C4—H4	122.1
N1—C1—C5	126.5 (5)	C7—S1—Hg1	99.9 (2)
N1—C1—C1^i^	124.4 (3)	O1—C6—C5^i^	127.5 (4)
C5—C1—C1^i^	109.1 (3)	O1—C6—C5	127.5 (4)
N1—C2—C3	122.9 (6)	C5^i^—C6—C5	105.0 (7)
N1—C2—H2	118.5		
			
S1^i^—Hg1—N1—C1	97.7 (3)	N1—C1—C5—C6	−179.7 (4)
S1—Hg1—N1—C1	−108.1 (3)	C1^i^—C1—C5—C6	−0.8 (6)
N1^i^—Hg1—N1—C1	−0.3 (2)	N1—C2—C3—C4	−1.1 (9)
S1^i^—Hg1—N1—C2	−82.2 (5)	C1—C5—C4—C3	0.0 (8)
S1—Hg1—N1—C2	71.9 (5)	C6—C5—C4—C3	179.5 (5)
N1^i^—Hg1—N1—C2	179.8 (6)	C2—C3—C4—C5	0.6 (9)
C2—N1—C1—C5	−0.5 (8)	S1^i^—Hg1—S1—C7	150.1 (3)
Hg1—N1—C1—C5	179.5 (4)	N1^i^—Hg1—S1—C7	−69.2 (3)
C2—N1—C1—C1^i^	−179.2 (6)	N1—Hg1—S1—C7	7.5 (3)
Hg1—N1—C1—C1^i^	0.8 (7)	C4—C5—C6—O1	0.7 (7)
C1—N1—C2—C3	1.1 (8)	C1—C5—C6—O1	−179.7 (3)
Hg1—N1—C2—C3	−179.0 (4)	C4—C5—C6—C5^i^	−179.3 (7)
N1—C1—C5—C4	0.0 (8)	C1—C5—C6—C5^i^	0.3 (3)
C1^i^—C1—C5—C4	178.9 (5)		

Symmetry codes: (i) −*x*+1, *y*, −*z*+1/2.

### Hydrogen-bond geometry (Å, °)

**Table:**

*D*—H···*A*	*D*—H	H···*A*	*D*···*A*	*D*—H···*A*
C2—H2···N2^ii^	0.93	2.56	3.276 (10)	134
C4—H4···O1^iii^	0.93	2.59	3.366 (8)	142

Symmetry codes: (ii) −*x*+1, *y*, −*z*−1/2; (iii) −*x*+1, −*y*+1, −*z*.

## References

[bb1] Biju, A. R. & Rajasekharan, M. V. (2008). *Polyhedron*, **27**, 2065–2068.

[bb2] Farrugia, L. J. (1997). *J. Appl. Cryst.* **30**, 565.

[bb3] Farrugia, L. J. (1999). *J. Appl. Cryst.* **32**, 837–838.

[bb4] Henderson, L. J. Jr, Fronczek, F. R. & Cherry, W. R. (1984). *J. Am. Chem. Soc.* **106**, 5876–5879.

[bb5] Kulkarni, P., Padhye, S., Sinn, E., Anson, C. E. & Powell, A. K. (2002). *Inorg. Chim. Acta*, **332**, 167–175.

[bb6] Menon, S. & Rajasekharan, M. V. (1998). *Polyhedron*, **17**, 2463–2476.

[bb7] Ravikumar, K. & Lakshmi, N. V. (1994). *Z. Kristallogr.* **209**, 56–57.

[bb8] Safari, N., Amani, V., Abedi, A., Notash, B. & Ng, S. W. (2009). *Acta Cryst.* E**65**, m372. 10.1107/S1600536809006904 PMC2969049 21582326

[bb9] Sheldrick, G. M. (2008). *Acta Cryst.* A**64**, 112–122. 10.1107/S0108767307043930 18156677

[bb10] Shi, X.-H., You, X.-Z., Li, C. & Xiong, R.-G. (1995). *Transition Met. Chem.* **20**, 191–195.

[bb11] Stoe & Cie (2005). *X-AREA*, *X-SHAPE* and *X-RED32* Stoe & Cie, Darmstadt, Germany.

[bb12] Wu, Z.-Y. & Xu, D.-J. (2004). *Acta Cryst.* E**60**, m839–m841.

[bb13] Zhang, R.-L., Zhao, J.-S., Yang, S.-Y. & Ng, S. W. (2004). *Acta Cryst.* E**60**, m262–m263.

